# Evaluation of Humoral Immune Response after SARS-CoV-2 Vaccination Using Two Binding Antibody Assays and a Neutralizing Antibody Assay

**DOI:** 10.1128/Spectrum.01202-21

**Published:** 2021-11-24

**Authors:** Minjeong Nam, Jong Do Seo, Hee-Won Moon, Hanah Kim, Mina Hur, Yeo-Min Yun

**Affiliations:** a Department of Laboratory Medicine, Korea University Anam Hospital, Seoul, South Korea; b Department of Laboratory Medicine, Konkuk University grid.258676.8grid.411120.7grid.258676.8 School of Medicine, Seoul, South Korea; UC Davis

**Keywords:** SARS-CoV-2, BNT162b2, ChAdOx1 nCoV-19, humoral immune response

## Abstract

Multiple vaccines against severe acute respiratory syndrome coronavirus 2 (SARS-CoV-2) have been developed and administered to mitigate the coronavirus disease 2019 (COVID-19) pandemic. We assessed the humoral response of BNT162b2 and ChAdOx1 nCoV-19 using Siemens SARS-CoV-2 IgG (sCOVG; cutoff of ≥1.0 U/ml), Abbott SARS-CoV-2 IgG II Quant (CoV-2 IgG II; cutoff of ≥50.0 AU/ml), and GenScript cPASS SARS-CoV-2 neutralization antibody detection kits (cPASS; cutoff of ≥30% inhibition). We collected 710 serum samples (174 samples after BNT162b2 and 536 samples after ChAdOx1 nCoV-19). Venous blood was obtained 3 weeks after first and second vaccinations. In both vaccines, sCOVG, CoV-2 IgG II, and cPASS showed a high seropositive rate (>95.7%) except for cPASS after the first vaccination with ChAdOx1 nCoV-19 (68.8%). Using sCOVG and CoV-2 IgG II, the ratios of antibody value (second/first) increased 10.6- and 11.4-fold in BNT162b2 (first 14.1, second 134.8 U/ml; first 1,416.2, second 14,326.4 AU/ml) and 2.3- and 2.0-fold in ChAdOx1 nCoV-19 (first 4.0, second 9.1 U/ml; first 431.0, second 9,744.0 AU/ml). cPASS-positive results indicated a very high concordance rate with sCOVG and CoV-2 IgG II (>98%), whereas cPASS-negative results showed a relatively low concordance rate (range of 22.2% to 66.7%). To predict cPASS positivity, we suggested additional cutoffs for sCOVG and CoV-2 IgG II at 2.42 U/ml and 284 AU/ml, respectively. In conclusion, BNT162b2 and ChAdOx1 nCoV-19 evoked robust humoral responses. sCOVG and CoV-2 IgG II showed a very strong correlation with cPASS. sCOVG and CoV-2 IgG II may predict the presence of neutralizing antibodies against SARS-CoV-2.

**IMPORTANCE** The Siemens severe acute respiratory syndrome coronavirus 2 (SARS-CoV-2) IgG (sCOVG; Siemens Healthcare Diagnostics Inc., NY, USA) and Abbott SARS-CoV-2 IgG II Quant (CoV-2 IgG II; Abbott Laboratories, Sligo, Ireland), which are automated, quantitative SARS-CoV-2-binding antibody assays, have been recently launched. This study aimed to evaluate the humoral immune response of BNT162b2 and ChAdOx1 nCoV-19 vaccines using sCOVG and CoV-2 IgG II and compare the quantitative values with the results of the GenScript surrogate virus neutralization test (cPASS; GenScript, USA Inc., NJ, USA). Our findings demonstrated that both BNT162b2 and ChAdOx1 nCoV-19 elicited a robust humoral response after the first vaccination and further increased after the second vaccination. sCOVG and CoV-2 IgG II showed a strong correlation, and the concordance rates among sCOVG, CoV-2 IgG II, and cPASS were very high in the cPASS-positive results. The additional cutoff sCOVG and CoV-2 IgG II could predict the results of cPASS.

## INTRODUCTION

Severe acute respiratory syndrome coronavirus 2 (SARS-CoV-2) causes coronavirus disease 2019 (COVID-19) (1). Physical distancing, face masks, and eye protection to prevent person-to-person transmission of SARS-CoV-2 cannot stop the COVID-19 pandemic alone (2). Thus, the development of SARS-CoV-2 vaccines is imperative to prevent further disease progression and severe mortality and morbidity (3). In addition, SARS-CoV-2 vaccines could terminate the COVID-19 pandemic by achieving herd immunity (4). Multiple vaccines have been developed for emergency clinical use or marketing based on multiple technologies, such as inactivated vaccines, viral vector vaccines, and mRNA vaccines (https://www.who.int/publications/m/item/draft-landscape-of-covid-19-candidate-vaccines). According to the Korean government’s guidelines for vaccination, 20.1% of individuals (10,399,289/51,821,669 on 2021 July 13) were vaccinated with ChAdOx1 nCoV-19, and 7.8% individuals (4,027,012/51,812,669 on 2021 July 13) were vaccinated with BNT162b2 (https://www.korea.kr/news/pressReleaseView.do?newsId=156461314).

Antibody testing against SARS-CoV-2 is helpful in predicting the prevalence of COVID-19, promptly diagnosing nonsymptomatic patients, and evaluating patients with COVID-19 after treatment (5). In the vaccination era of the COVID-19 pandemic, another role of antibody testing is to monitor the presence of antibodies against SARS-CoV-2 after vaccination (6). The antibody test is broadly classified as either binding or neutralizing antibody assays. Binding antibody assays can detect antibodies (IgG, IgM, or total) against the spike protein receptor-binding domain (RBD), partial spike protein (S1 subunit, S2 subunit), or nucleocapsid protein (N) (7). The neutralizing antibody assay determines the presence of functional antibodies to prevent SARS-CoV-2 infection. (8). Thus, many researchers have developed new assays to measure neutralizing antibodies that can be performed in clinical laboratories within a few hours, such as the GenScript surrogate virus neutralization test (cPASS; GenScript, USA Inc., NJ, USA) (9, 10). Many studies have evaluated the performance of SARS-CoV-2-binding antibody assays in COVID-19 patients with different target antigens and methodologies (7, 11–14). Previous studies have reported that assays targeting the RBD and detecting IgG showed the highest sensitivity and specificity (7, 12). In the plasma of SARS-CoV-2 patients, the SARS-CoV-2 IgG-binding antibody assay and neutralizing antibody assay were highly correlated (11, 13, 14). Although noninfected individuals with SARS-CoV-2 are more prevalent in the global population, few studies on the humoral immune response of noninfected individuals after vaccination have been conducted.

The Siemens SARS-CoV-2 IgG (sCOVG; Siemens Healthcare Diagnostics Inc., NY, USA) and Abbott SARS-CoV-2 IgG II Quant (CoV-2 IgG II; Abbott Laboratories, Sligo, Ireland), which are quantitative SARS-CoV-2-binding antibody assays, have been recently launched. This study aimed to evaluate the humoral immune response of BNT162b2 and ChAdOx1 nCoV-19 vaccines using sCOVG and CoV-2 IgG II and compare the quantitative values of sCOVG and CoV-2 IgG II with the results of cPASS.

## RESULTS

### Results of three assays after first and second vaccination of BNT162b2 and ChAdOx1 nCoV-19.

After the first and second vaccinations of BNT162b2 mRNA and ChAdOx1 nCoV-19, high seropositive rates were observed (>95.7%) in sCOVG, CoV-2 IgG II, and cPASS, except cPASS after the first vaccination with ChAdOx1 nCoV-19 (68.8%) (Table 1). In BNT162b2, the median antibody values after the second vaccination showed a 10.6- and 11.4-fold increase in sCOVG and CoV-2 IgG II compared to the first vaccination (sCOVG: first 14.1, second 134.8 U/ml; CoV-2 IgG II: first 1,416.2, second 14,326.4 AU/ml). In ChAdOx1 nCoV-19, the median antibody values after the second vaccination showed a 2.3- and 2.0-fold increase compared to the first vaccination (sCOVG: first 4.0, second 9.1 U/ml; CoV-2 IgG II: first 431.0, second 974.4 AU/ml) (Table 2). The female group showed slightly higher antibody values than the male group after the first vaccination but did not show a significant difference after the second vaccination (data not shown). The differences among age groups were not statistically significant (*P > *0.05) (Table 2).

**TABLE 1:** Seropositive rates of sCOVG, CoV-2 IgG II, and cPASS after BNT162b2 and ChAdOx1 nCoV-19 vaccination

Assay^*a*^	BNT162b2				ChAdOx1 nCoV-19		
	First vaccination	Second vaccination	*P*		First vaccination	Second vaccination	*P*
*n*	88	86			278	258	
Siemens sCOVG							
Positive, *n* (%)	87 (98.9)	86 (100)	0.33		247 (88.8)	256 (99.2)	<0.001
Negative, *n* (%)	1 (1.1)	0 (0)			31 (11.2)	2 (0.8)	
Abbott, CoV-2 IgG II							
Positive, *n* (%)	87 (98.9)	86 (100)	0.33		266 (95.7)	256 (99.2)	0.01
Negative, *n* (%)	1 (1.1)	0 (0)			12 (4.3)	2 (0.8)	
*n*	88	86			112	256	
GenScript, cPASS							
Positive, *n* (%)	85 (96.6)	86 (100)	0.09		77 (68.8)	249 (97.3)	<0.001
Negative, *n* (%)	3 (3.4)	0 (0)			35 (31.2)	7 (2.7)	

a *n*, number; sCOVG, SARS-CoV-2 IgG; CoV-2 IgG II, SARS-CoV-2 IgG II Quant; cPASS, cPASS SARS-CoV-2 neutralization antibody detection.

**TABLE 2:** SARS-CoV-2 IgG response after BNT162b2 and ChAdOx1 nCoV-19 vaccination

Assay^*a*^	Age group (yrs)^*a*^	BNT162b2					
		First vaccination (*n *= 88)^*b*^	*P*	Second vaccination (*n *= 86)^*b*^	*P*	Second/first ratio (*n *= 86)^*b*^	*P*
Siemens, sCOVG	20–29, median (IQR)	13.6 (7.9–23.5)	0.19	150.0 (94.7–266.8)	0.08	11.8 (5.9–16.9)	0.76
	30–39, median (IQR)	13.2 (8.2–24.0)		133.3 (95.8–225.0)		10.3 (4.6–12.4)	
	40–49, median (IQR)	14.1 (7.9–20.8)		113.8 (85.0–155.7)		10.2 (5.3–16.5)	
	≥50, median (IQR)	16.1 (13.6–21.0)		132.9 (96.5–542.3)		9.8 (7.2–22.0)	
	Total, median (IQR)	14.1 (8.6–22.8)		134.8 (95.5–210.8)		10.6 (6.1–16.0)	
Abbott, CoV-2 IgG II	20–29, median (IQR)	1,475.8 (701.2–2,448.5)	0.23	16,016.8 (8,798.8–23,911.6)	0.07	10.3 (5.6–16.6)	0.38
	30–39, median (IQR)	1,423.8 (885.1–1,980.4)		15,420.9 (11,797.0–25,645.9)		10.2 (7.7–13.8)	
	40–49, median (IQR)	1,332.5 (904.7–8,085.4)		11,257.9 (8,085.4–17,826.8)		10.8 (5.5–14.7)	
	≥50, median (IQR)	1,654.5 (1,279.1–2,286.6)		14,849.1 (12,571.9–37,190.8)		13.6 (8.0–17.0)	
	Total, median (IQR)	1,416.2 (877.9–2,090.8)		14,326.4 (9,986.3–21,076.3)		11.4 (6.7–14.8)	
		**ChAdOx1 nCoV-19**					
**Assay**	**Age group (yrs)**	**First vaccination** **(*n *= 278)**	***P***	**Second vaccination** **(*n *= 258)**	***P***	**Second/first ratio** **(*n *= 244)**	***P***
Siemens, sCOVG	20–29, median (IQR)	5.5 (2.3–10.6)	0.69	10.3 (6.7–10.7)	0.41	1.9 (0.9–4.3)	0.30
	30–39, median (IQR)	4.3 (2.0–10.0)		10.6 (6.6–18.3)		2.0 (1.1–6.0)	
	40–49, median (IQR)	3.0 (1.1–7.6)		9.5 (5.0–18.7)		3.0 (1.2–7.1)	
	≥50, median (IQR)	3.6 (2.0–7.0)		7.4 (3.8–18.7)		1.8 (1.0–7.3)	
	Total, median (IQR)	4.0 (1.9–9.5)		9.4 (5.9–19.7)		2.3 (1.1–5.9)	
Abbott, CoV-2 IgG II	20–29, median (IQR)	571.0 (255.6–1,060.5)	0.86	1,095.6 (701.6–1,796.7)	0.37	1.8 (1.0–3.6)	0.31
	30–39, median (IQR)	490.8 (226.1–947.1)		1,095.5 (703.2–1,555.2)		1.8 (1.0–4.3)	
	40–49, median (IQR)	328.6 (131.2–893.1)		915.8 (533.6–1,616.7)		2.7 (1.2–7.1)	
	≥50, median (IQR)	431.0 (242.0–617.5)		809.3 (448.1–1,497.8)		2.0 (1.1–5.6)	
	Total, median (IQR)	431.0 (212.9–952.0)		974.4 (604.0–1,596.5)		2.0 (1.1–5.2)	

a *n*, number; IQR, interquartile range; sCOVG, SARS-CoV-2 IgG; CoV-2 IgG II, SARS-CoV-2 IgG II Quant.

b Data are shown as median (interquartile range).

### Qualitative concordance among two binding antibody assays and a neutralizing antibody assay.

The concordance rates and agreement between the two binding antibody assays and the neutralizing antibody assay in vaccinated individuals are shown in Table 3. The cPASS-positive results showed a 100% concordance rate with sCOVG and CoV-2 IgG II in the current cutoff (≥30% inhibition) and 98.1% and 99.6% concordance rates in the previous cutoff (≥20% inhibition). cPASS-negative results in the current cutoff (≥30% inhibition) showed relatively lower concordance rates than those in the previous cutoff (≥20 inhibition) (≥30% versus ≥20%: 48.9% versus 66.7% at sCOVG; 22.2% versus 44.4% at CoV-2 IgG II) (Table 3). Agreement between sCOVG and cPASS was strong in both cutoffs (Cohen’s kappa [*κ*] = 0.80 at ≥30%; *κ* = 0.82 at ≥20%), and that between CoV-2 IgG II and cPASS was moderate to strong (*κ* = 0.67 at ≥30%; *κ* = 0.83 at ≥20%).

**TABLE 3:** Concordance rate and agreement between two binding antibody assays and a neutralizing antibody assay

Assay^*a*^	Current cutoff (≥30% inhibition)		Kappa (CI)
	cPASS positive^*a*^ (*n *= 496)	cPASS negative (*n *= 45)	
Siemens, sCOVG			
Positive, *n* (%)	496 (100.0)	23 (51.1)	0.80 (0.73–0.87)
Negative, *n* (%)	0 (0.0)	22 (48.9)	
Abbott, CoV-2 IgG II			
Positive, *n* (%)	496 (100.0)	35 (77.8)	0.67 (0.58–0.77)
Negative, *n* (%)	0 (0.0)	10 (22.2)	
	**Previous cutoff (≥20% inhibition)**		
**Assay**	**cPASS positive** **(*n *= 523)**	**cPASS negative** **(*n *= 18)**	**Kappa (CI)**
Siemens, sCOVG			
Positive, *n* (%)	513 (98.1)	6 (33.3)	0.82 (0.74–0.90)
Negative, *n* (%)	10 (1.9)	12 (66.7)	
Abbott, CoV-2 IgG II			
Positive, *n* (%)	521 (99.6)	10 (55.6)	0.83 (0.75–0.91)
Negative, *n* (%)	2 (0.4)	8 (44.4)	

a cPASS, cPASS SARS-CoV-2 neutralization antibody detection; sCOVG, SARS-CoV-2 IgG; CoV-2 IgG II, SARS-CoV-2 IgG II Quant; *n*, number.

### Quantitative relations among two binding antibody assays and a neutralizing antibody assay.

**FIG 1:**
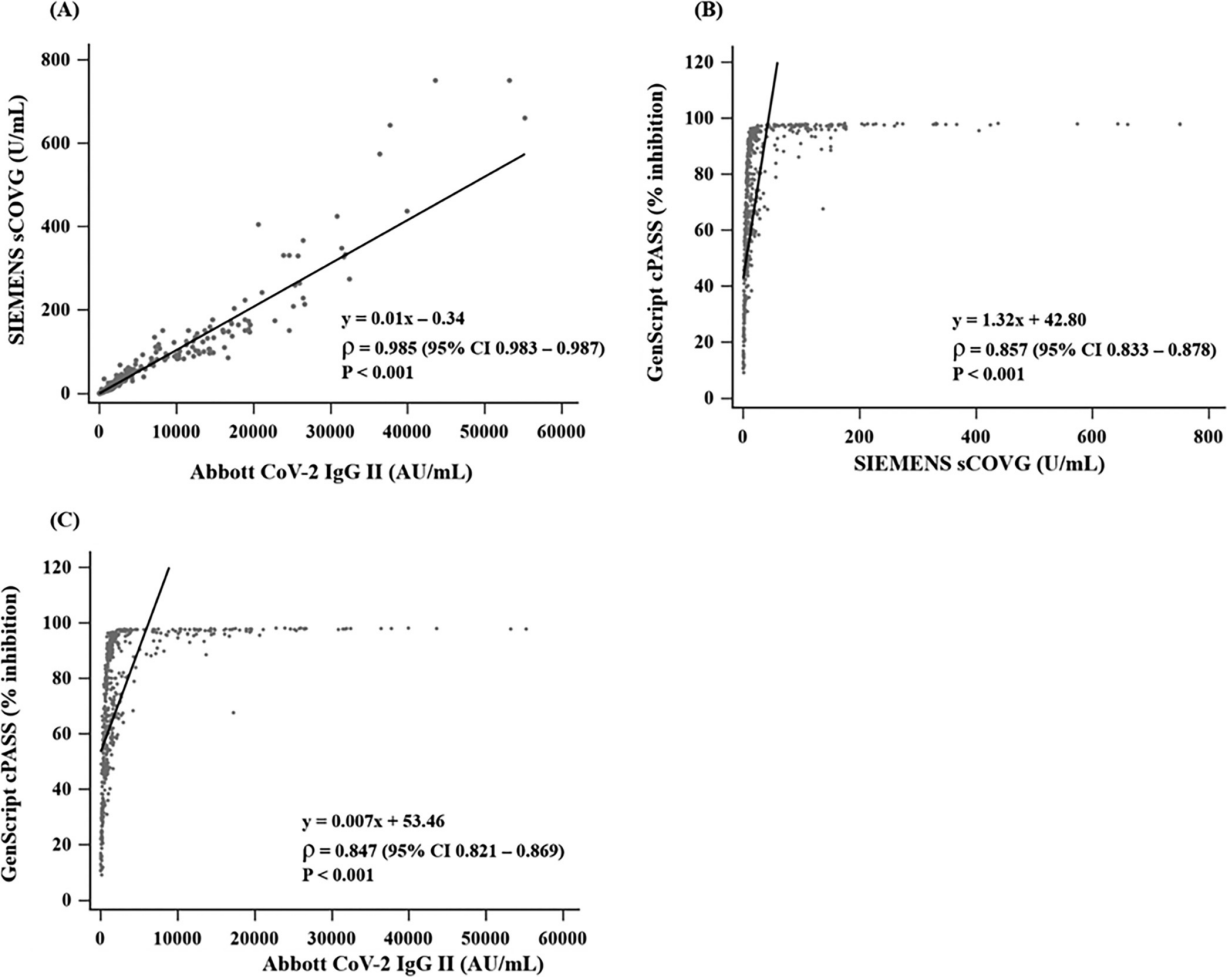
Quantitative correlation among two antibody-binding assays and a neutralizing antibody assay. Using Passing-Bablok regression and Spearman correlation, (A) sCOVG (U/ml) and CoV-2 IgG II were compared, and (B) sCOVG (U/ml) and (C) CoV-2 IgG II (AU/ml) were compared with cPASS. The black line represents the regression line.

**FIG 2:**
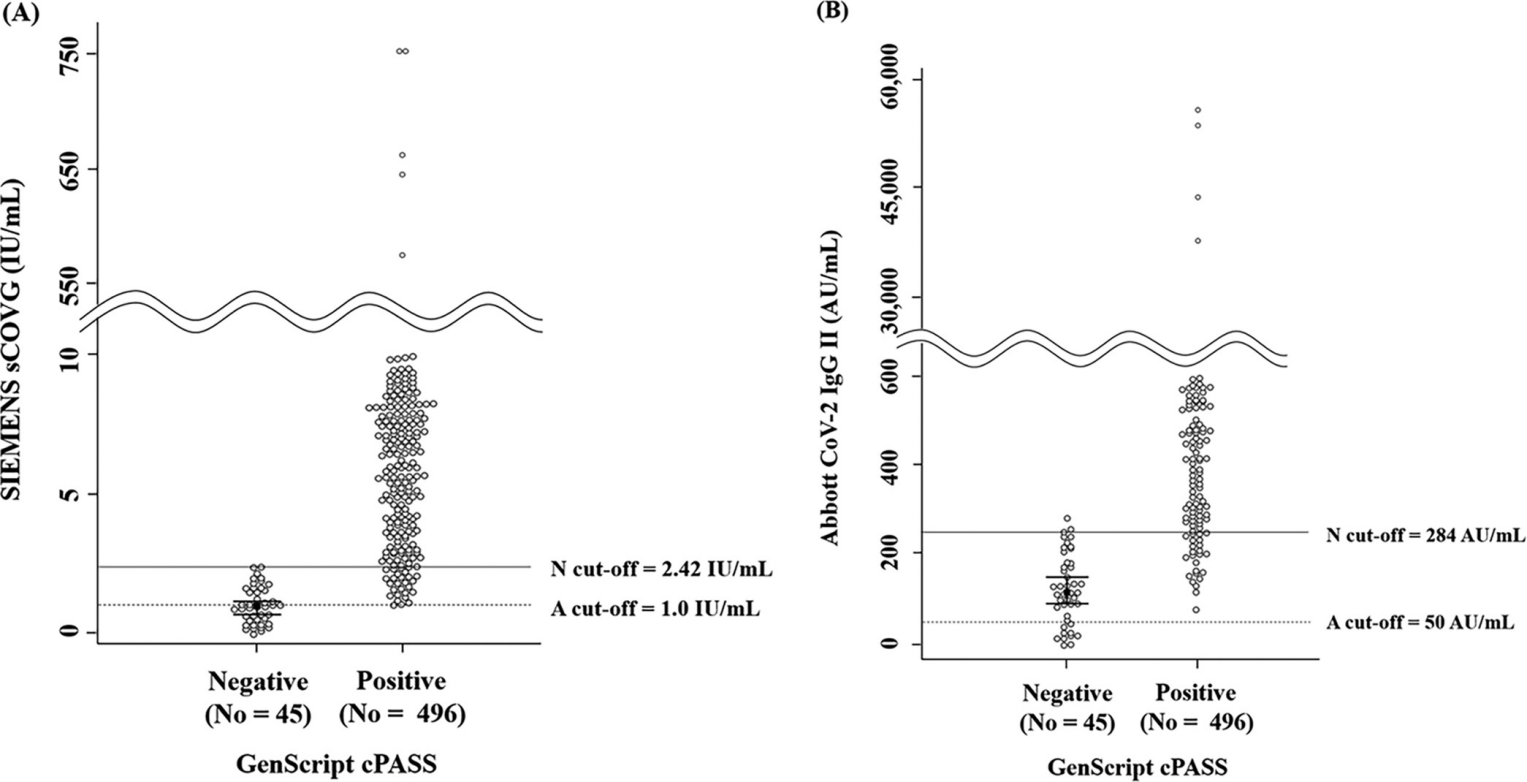
Comparison of antibody values for two antibody-binding assays based on the results of a neutralizing antibody assay. (A) sCOVG (U/ml). The median value of sCOVG at cPASS-negative results was 1.0 U/ml (0.5 to 1.7), and that of cPASS-positive results was 12.9 U/ml (IQR 6.7 to 30.1). (B) CoV-2 IgG II (AU/ml). The median value of CoV-2 IgG II at cPASS-negative results was 117.6 AU/ml (IQR of 65.3 to 184.5), and that of cPASS-positive results was 1,266.1 AU/ml (IQR of 689.2 to 2,687.3). The solid line indicates the cutoff to predict neutralizing antibodies, and the dashed line indicates the cutoff claimed by manufacturers to predict antibodies against SARS-CoV-2. Abbreviations: N cutoff, cutoff for neutralizing antibodies; A cutoff, cutoff for antibodies against SARS-CoV-2 from manufacturers.

A very high correlation was shown when comparing quantitative values between sCOVG and CoV-2 IgG II (Spearman’s correlation coefficient [*ρ*] = 0.985 [confidence interval (CI), 0.983 to 0.987], *P < *0.001). When we compared the values of sCOVG and CoV-2 IgG II with cPASS (percent inhibition), sCOVG and CoV-2 IgG II showed moderate correlations with cPASS (*ρ* = 0.857 [CI, 0.833 to 0.878], *P < *0.001 and *ρ* = 0.847 [CI, 0.821 to 0.869], *P < *0.001), respectively (Fig. 1). In the cPASS-negative results, the median antibody values were 1.0 U/ml (interquartile range [IQR] of 0.5 to 1.7) in sCOVG and 117.6 AU/ml (IQR of 65.3 to 184.5) in CoV-2 IgG II. In the cPASS-positive results, the median antibody values were 12.9 U/ml [IQR of 6.7 to 30.1] in sCOVG and 1,266.1 AU/ml (IQR of 689.2 to 2,687.3) in CoV-2 IgG II. The optimized cutoff value to predict cPASS positivity (≥30% inhibition) was 2.42 U/ml in sCOVG and 284 AU/ml in CoV-2 IgG II (Fig. 2). At this cutoff, the sensitivity and specificity for predicting cPASS were 94.6% and 97.8% in sCOVG and 93.6% and 97.8% in CoV-2 IgG II, respectively.

## DISCUSSION

Our results demonstrated that robust seropositive rates were observed after the first and second vaccinations of BNT162b2 and ChAdOx1 nCoV-19 using sCOVG and CoV-2 IgG II. Corresponding well with these findings, an earlier study reported that seropositive rates after first and second vaccinations of both vaccines were very high using CoV-2 IgG II (BNT162b2 first 228/230, 99% and second 82/82, 100%; ChAdOx1 nCoV-19 first 82/87, 94% and second 2/2, 100%) (15). Conversely, other studies reported relatively low seropositive rates, which were 75.4% using sCOVG after the first vaccination with ChAdOx1 nCoV-19 vaccine and 85.2% and 89.3% using CoV-2 IgG II after the first vaccination with BNT162b2 and ChAdOx1 nCoV-19 vaccines (16, 17). The seropositive rates were significantly different according to antibody assays (66.2 to 92.5%) (14), which may be attributed to variance of assay sensitivities with different target antigens and methodologies. In addition, manufacturers adjust the assays and launch a new version of their assays.

Considering quantitative results after vaccination, in a previous study using CoV-2 IgG II (15), the median antibody values were 1,028 AU/ml (564 to 1,685) and 10,058 AU/ml (6,408 to 15,582) at the first and second vaccination of BNT162b2 and 435 AU/ml (203 to 962) at the first vaccination of ChAdOx1 nCoV-19, similar to our quantitative data. According to the previous study (15), BNT162b2 resulted in higher antibody values than ChAdOx1 nCoV-19, although the seropositive rates between both vaccines were similar (2.4- or 3.3-fold higher at the first vaccination; 14.7-fold higher at the second vaccination). Another previous study using Roche Elecsys SARS-CoV-2 total antibody could not be compared directly, but they also demonstrated that BNT162b2 showed higher values and higher fold increase (1.4-fold higher at the first vaccination; 2.2-fold higher at the second vaccination) than ChAdOx1 nCoV-19 (18). However, a higher value of SARS-CoV-2 antibodies after vaccination should not be considered evidence of a more effective vaccine. Quantitative values of antibodies could be associated with neutralizing activity, but the proper level for protection from infection and disease severity remains unknown (19, 20). The humoral immune response after vaccination could differ according to age, gender, and ethnicity. This study demonstrated that age and gender did not statistically affect the antibody response after the second vaccination, and other previous studies showed conflicting results for vaccine response by age and gender (14, 15, 18, 21). Moreover, the dosing interval of BNT162b2 is 3 to 4 weeks whereas that of ChAdOx1 nCoV-19 is 8 to 12 weeks to maximize the effect of the first vaccination (https://www.gov.uk/government/publications/prioritising-the-first-covid19-vaccine-dose-jcvi-statement/optimising-the-covid-19-vaccination-program-for-maximum-short-termimpact). The antibody values in both BNT162b2 and ChAdOx1 nCoV-19 declined over time with different rates after the first vaccination during different periods (22); the quantitative values in both vaccines cannot be compared directly.

The most critical determinant for producing a high value of SARS-CoV-2 antibodies is previous infection (23). In this study, one patient was infected with SARS-CoV-2 after the first vaccination with ChAdOx1 nCoV-19, even though the case was excluded from the analysis. After the second vaccination, sCOVG and CoV-2 IgG II showed 223.26 U/ml and 18,889.6 AU/ml, which were the maximum values of this study population and were 23.8- and 19.4-fold higher than the median value. Similar to this study, many previous studies demonstrated that the humoral immune response after the first vaccination was 2- to 26.7-fold higher in previously infected individuals than in noninfected individuals (24, 25).

The plaque reduction neutralization test (PRNT) is capable of quantifying the level of neutralizing antibodies against SARS-CoV-2. However, this assay is time-consuming and labor-intensive and requires biosafety laboratory level 3 facilities to work with the risk group 3 pathogen; it causes the limited capacity to be implemented in high-throughput tests. cPASS, however, is a commercially available assay that quantifies inhibition of the RBD-angiotensin converting enzyme 2 (ACE2) interaction without the use of live viruses. cPASS demonstrated similar detection rates and percent inhibition value that correlated well with PRNT-50 and PRNT-90 (9, 26). In addition, the cross-reactivity of cPASS for SARS-CoV-1 was approximately 70%, which was significantly higher than PRNT-50 and PRNT-90 (9, 26). In the past, there were three infected cases with SARS-CoV-1 in South Korea, so it seems not to consider cross-reactivity with SARS-CoV-1 (https://www.who.int/publications/m/item/summary-of-probable-sars-cases-with-onset-of-illness-from-1-november-2002-to-31-july-2003).

The WHO has recently cautioned that positive results of binding antibody assays do not ensure the presence of neutralizing antibodies (https://www.who.int/emergencies/diseases/novel-coronavirus-2019/media-resources/science-in-5/episode-14---covid-19---tests?gclid=Cj0KCQjwxdSHBhCdARIsAG6zhlWCaF98SFZCRTwPsl1sCRYA2imH0jyQSXvnkRUhsYTQPWXeWWK0kFYaAuKoEALw_wcB). To evaluate humoral immune response after vaccination, it should be prioritized to select a valuable binding antibody assay that correlates with the neutralizing antibody assay (27). In this study, the positive results of sCOVG and CoV-2 IgG II showed very strong concordance rates with cPASS-positive results at both current and previous cutoff values. However, the negative results of sCOVG and CoV-2 IgG II indicated lower concordance rates at the current cPASS cutoff than at the previous cutoff. The agreements between binding antibody assays and neutralizing antibody assay at the current cutoff were lower (*κ* = 0.80 and 0.67) than the previous cutoff (*κ* = 0.82 and 0.83). The critical cutoff should be established over many years from a standard assay that provides specific data about 50% protection from SARS-CoV-2 infection (9, 20). However, there are few standardized assays for assessing the neutralizing activity of SARS-CoV-2 and little data comparing specific levels of neutralizing antibodies to protect against SASR-CoV-2 infection. Further studies are needed to elucidate the relationship between neutralizing antibody levels and protection against infection.

sCOVG and CoV-2 IgG II targeting RBD showed some qualitative discrepancy around the cutoff, but their quantitative values showed a very high correlation (*ρ* = 0.985, CI 0.983 to 0.987, *P < *0.001). The determination of cutoff is critical and should be standardized in the future. Although cPASS is not intended for use as a quantitative assay, quantitative results of sCOVG and CoV-2 IgG II were highly correlated with cPASS in this study (sCOVG: *ρ* = 0.857, CI 0.833 to 0.878, *P < *0.001; CoV-2 IgG: *ρ* = 0.847, CI 0.821 to 0.869, *P < *0.001, respectively). Previous studies also demonstrated a good qualitative agreement of sCOVG and CoV-2 IgG II with cPASS and better correlation in S protein-based antibody-binding assays than N protein-based assays (11, 28, 29). Moreover, we suggested an additional cutoff 2.42 U/ml for sCOVG and 284 AU/ml for CoV-2 IgG II to predict cPASS positivity. The previous study investigated the good performance of sCOVG and CoV-2 IgG II for predicting neutralizing antibodies, but they did not suggest a definite cutoff (28). Thus, although the binding antibody assay is intended to detect IgG, IgM, or total antibodies against SARS-CoV-2, we suggested the cutoff level of antibody predicting the positive neutralizing antibody to help verify the effective humoral response after vaccination.

This study has some limitations. In this study, we could not include individuals over 65 years of age and individuals with a severe underlying disease that elicit a different immune response than healthy individuals. Further studies should validate the humoral response of old age and individuals with severe underlying diseases. Nevertheless, most people who were vaccinated were healthy individuals; thus, this study is also meaningful. Additionally, this study did not include baseline data for prevaccination. However, the seroprevalence of SARS-CoV-2 IgG was very low in South Korea during the study design and sample collection period, and data in our institution showed very low positivity in community and health care workers (0.0% and 0.6%, respectively) (30).

In conclusion, our findings demonstrated that both BNT162b2 and ChAdOx1 nCoV-19 elicited a robust humoral response after the first vaccination and further increased after the second vaccination. sCOVG and CoV-2 IgG II showed a strong correlation, and the concordance rates among sCOVG, CoV-2 IgG II, and cPASS were very high in the cPASS-positive results. The additional cutoff sCOVG and CoV-2 IgG II could predict the presence of neutralizing antibodies.

## MATERIALS AND METHODS

### Study population.

This study included 710 serum samples from 379 subjects (174 serum samples from 88 subjects after BNT162b2 vaccination and 536 serum samples from 291 subjects after ChAdOx1 nCoV-19 vaccination) from March to June 2021 at Konkuk University Medical Center (KUMC) in South Korea. All subjects were ≥18 years of age and provided written informed consent before enrollment. Subjects were excluded if they (i) were less than 18 years old, (ii) had a history of SARS-CoV-2 infection, (iii) were pregnant, or (iv) missed sampling day 3 weeks after first and second vaccinations. This study was approved by the Institutional Review Board of KUMC (institutional review board number 2021-03-015-003). According to the government’s guidelines for SARS-CoV-2 vaccination, the dosing interval of BNT162b2 is 3 weeks, and that of ChAdOx1 nCoV-19 ranges from 8 weeks to 12 weeks (https://www.korea.kr/news/pressReleaseView.do?newsId=156461314). Three weeks after the first and second vaccinations of BNT162b2 and ChAdOx1 nCoV-19, venous blood samples of all subjects were collected into Vacuette CAT serum clot activator (Greiner Bio-One, GmbH, Kremsmunster, Austria). After immediate centrifugation at 1,977 × *g* for 10 min, each sample was aliquoted into two tubes and stored at 80°C. All samples were divided into the following four groups according to age: (i) 20 to 29 (*n* = 211), (ii) 30 to 39 (*n* = 195), (iii) 40 to 49 (*n* = 196), and (iv) ≥50 (*n* = 108). Females were prevalent in all age groups (range of 58.7% to 90.7%) (Table 4).

**TABLE 4:** Characteristics of the study population

Age group (yrs)	Parameters^*a*^	BNT162b2		ChAdOx1 nCoV-19	
		First vaccination	Second vaccination	First vaccination	Second vaccination
20–29	*n*	35	34	75	67
	Male, *n* (%)	6 (17.1)	5 (14.7)	7 (9.3)	7 (10.4)
	Female, *n* (%)	29 (82.9)	29 (85.3)	68 (90.7)	60 (89.6)
30–39	*n*	23	22	77	73
	Male, *n* (%)	4 (17.4)	4 (18.2)	10 (13.0)	9 (12.3)
	Female, *n* (%)	19 (82.6)	18 (81.8)	67 (87.0)	64 (87.7)
40–49	*n*	21	21	80	74
	Male, *n* (%)	3 (14.3)	3 (14.3)	26 (32.5)	23 (31.1)
	Female, *n* (%)	18 (85.7)	18 (85.7)	54 (67.5)	51 (68.9)
≥50	*n*	9	9	46	44
	Male, *n* (%)	2 (22.2)	2 (22.2)	19 (41.3)	18 (40.9)
	Female, *n* (%)	7 (77.8)	7 (77.8)	27 (58.7)	26 (59.1)
Total	*n*	88	86	278	258
	Male, *n* (%)	15 (17.0)	14 (16.3)	62 (22.3)	57 (22.1)
	Female, *n* (%)	73 (83.0)	72 (83.7)	216 (77.7)	201 (77.9)

a *n*, number.

### SARS-CoV-2 IgG antibody assays.

The 710 serum samples were tested using sCOVG and CoV-2 IgG II according to the manufacturer’s instructions. The sCOVG and CoV-2 IgG II are automated two-step sandwich antibody-binding immunoassays using indirect chemiluminescent technology. sCOVG and CoV-2 IgG II were used for qualitative and quantitative detection of IgG antibodies against the RBD of the S1 spike protein. sCOVG was interpreted as reactive (≥1.0 U/ml) or nonreactive (<1.0 U/ml). The analytical measurement interval (AMR) was 0.50 to 150.00 U/ml, and the results with over 150 U/ml were diluted and retested to obtain accurate results. CoV-2 IgG II was interpreted as positive (≥50.0 AU/ml) or negative (<50.0 AU/ml). AMR is 21.0 to 40,000.0 AU/ml, and the extended measuring interval (EMI) by dilution was 40,000.0 to 80,000.0 AU/ml.

cPASS was evaluated on 174 serum samples for BNT162b2 and 367 serum samples for ChAdOx1 nCoV-19 according to the manufacturer’s instructions. cPASS is a competitive enzyme-linked immunosorbent assay technology detecting the presence of neutralizing antibodies. The angiotensin-converting enzyme 2 (ACE2) receptor precoated on the microplate was incubated with horseradish peroxidase (HRP)-labeled RBD, which generated a strong signal. If neutralizing antibodies were present in the sample, the neutralizing antibody would bind to HRP-labeled RBD and prevent binding to the ACE2 receptor on the microplate. Serum samples with more neutralizing antibodies generate a low signal. The signal was recorded by the optical density (OD) at 450 nm, and the following formula was used to calculate the level of signal inhibition: signal inhibition (%) = (1 − OD value of sample/OD value of negative control) × 100. The results were interpreted as positive at ≥30% inhibition (9), which was changed from the 20% inhibition claimed by the manufacturer (31).

### Statistical analysis.

After testing distribution normality and homogenous variation using a Kolmogorov-Smirnov test, continuous variables with nonnormal distribution were expressed as the median (interquartile range). Categorical variables were expressed as numbers (percentages). Analyses of differences among age and gender groups were performed using analysis of variance (ANOVA). The agreement between two antibody-binding assays and a neutralizing antibody assay was evaluated by Cohen’s kappa with a 95% confidence interval (CI), which was interpreted as follows: ≤0.20, none; 0.21 to 0.39, minimal; 0.40 to 0.59, weak; 0.60 to 0.79, moderate; 0.80 to 0.90, strong; and >0.90, almost perfect (32). The quantitative values of each assay were assessed using Spearman correlation of rank correlation (*ρ*) and Passing-Bablok regression. The Spearman’s correlation coefficients (*ρ*) with a 95% CI were interpreted as follows: 0.00 to 0.30, negligible; 0.30 to 0.50, weak; 0.50 to 0.70, moderate; 0.70 to 0.90, high; 0.90 to 1.0, very high (33). The cutoff values of sCOVG and CoV-2 IgG II to predict cPASS positivity (≥30% inhibition) were calculated using the Youden maximum index value with equal weight to sensitivity and specificity. All statistical analyses were performed using MedCalc statistical software (version 19.4.0; MedCalc Software Bvba, Ostend, Belgium) and SPSS software (version 25.0; IBM Corp., 2014, Armonk, NY, USA).

### Data availability.

A data set of serological responses of 710 samples was deposited at https://dataverse.harvard.edu/ (https://doi.org/10.7910/DVN/RCIX8H).
